# MLST-Based Population Genetic Analysis in a Global Context Reveals Clonality amongst *Cryptococcus neoformans* var. *grubii* VNI Isolates from HIV Patients in Southeastern Brazil

**DOI:** 10.1371/journal.pntd.0005223

**Published:** 2017-01-18

**Authors:** Kennio Ferreira-Paim, Leonardo Andrade-Silva, Fernanda M. Fonseca, Thatiana B. Ferreira, Delio J. Mora, Juliana Andrade-Silva, Aziza Khan, Aiken Dao, Eduardo C. Reis, Margarete T. G. Almeida, Andre Maltos, Virmondes R. Junior, Luciana Trilles, Volker Rickerts, Ariya Chindamporn, Jane E. Sykes, Massimo Cogliati, Kirsten Nielsen, Teun Boekhout, Matthew Fisher, June Kwon-Chung, David M. Engelthaler, Marcia Lazéra, Wieland Meyer, Mario L. Silva-Vergara

**Affiliations:** 1 Molecular Mycology Research Laboratory, Centre for Infectious Diseases and Microbiology, Marie Bashir Institute for Emerging Infectious Diseases and Biosecurity, Sydney Medical School-Westmead Hospital, The Westmead Institute for Medical Research, The University of Sydney, Sydney, Australia; 2 Infectious Disease Department, Triangulo Mineiro Federal University, Uberaba, Brazil; 3 Biomedicine Department, Federal University of Piauí, Parnaíba, Brazil; 4 Infectious Disease Department, Faculty of Medicine of São José do Rio Preto, São José do Rio Preto, Brazil; 5 Evandro Chagas National Institute of Infectious Diseases, Oswaldo Cruz Foundation, Rio de Janeiro, Brazil; 6 Mycology Section, Robert Koch Institute, Berlin, Germany; 7 Mycology Unit, Department of Microbiology, Faculty of Medicine, Chulalongkorn University, Bangkok, Thailand; 8 Department of Medicine and Epidemiology, University of California, Davis, United States of America; 9 Laboratorio Micologia Medica, Dip. Scienze Biomediche per la Salute, Università degli Studi di Milano, Milano, Italy; 10 Department of Microbiology and Immunology, Medical School, University of Minnesota, Minneapolis, Mississippi, United States of America; 11 Department of Yeast and Basidiomycete Research, CBS-KNAW Fungal Biodiversity Centre, Utrecht, The Netherlands; 12 Department of Infectious Disease Epidemiology, Imperial College London, Norfolk Place, London, United Kingdom; 13 Molecular Microbiology Section, Laboratory of Clinical Infectious Diseases, National Institute of Allergy and Infectious Diseases, National Institute of Health, Bethesda, Maryland, United States of America; 14 Translational Genomics Research Institute, Flagstaff, Arizona, United States of America; University of California San Diego School of Medicine, UNITED STATES

## Abstract

Cryptococcosis is an important fungal infection in immunocompromised individuals, especially those infected with HIV. In Brazil, despite the free availability of antiretroviral therapy (ART) in the public health system, the mortality rate due to *Cryptococcus neoformans* meningitis is still high. To obtain a more detailed picture of the population genetic structure of this species in southeast Brazil, we studied 108 clinical isolates from 101 patients and 35 environmental isolates. Among the patients, 59% had a fatal outcome mainly in HIV-positive male patients. All the isolates were found to be *C*. *neoformans* var. *grubii* major molecular type VNI and mating type locus alpha. Twelve were identified as diploid by flow cytometry, being homozygous (AαAα) for the mating type and by PCR screening of the *STE20*, *GPA1*, and *PAK1* genes. Using the ISHAM consensus multilocus sequence typing (MLST) scheme, 13 sequence types (ST) were identified, with one being newly described. ST93 was identified from 81 (75%) of the clinical isolates, while ST77 and ST93 were identified from 19 (54%) and 10 (29%) environmental isolates, respectively. The southeastern Brazilian isolates had an overwhelming clonal population structure. When compared with populations from different continents based on data extracted from the ISHAM-MLST database (mlst.mycologylab.org) they showed less genetic variability. Two main clusters within *C*. *neoformans* var. *grubii* VNI were identified that diverged from VNB around 0.58 to 4.8 million years ago.

## Author Summary

The members of the *Cryptococcus neoformans* / *Cryptococcus gattii* species complex are the cause of cryptococcosis, a life-threatening human disease responsible for 624,000 deaths annually. Infection is acquired through inhalation of dehydrated yeast cells from environmental sources. After reaching the lungs, the fungus disseminates to the central nervous system causing meningoencephalitis. The majority of meningitis cases in HIV-infected patients are caused by *C*. *neoformans*, a species well studied in regions with a high prevalence of HIV infection, such as Asia and Africa. A similar high prevalence has been reported from Brazil however the epidemiology of these infections is less well understood. We studied clinical and environmental isolates from the southeast region of Brazil using MLST. The results that we obtained showed a clonal population structure of *C*. *neoformans* var. *grubii* VNI, with low variability when compared against populations from different continents. This lower variability is probably the result of multiple recent dispersal events from Africa to the Americas. The majority of clinical isolates were of one sequence type (ST93), which was also found in environmental samples. By expanding the analysis to isolates from around the globe, it was possible to identify two major groups among *C*. *neoformans* var. *grubii* VNI.

## Introduction

Infection by *Cryptococcus* species is considered one of the most important disease in patients living with HIV. It is estimated that around 624,000 deaths occur annually due to cryptococcosis, with most of them occurring in sub-Saharan Africa and Southeast Asia [1, 2]. The infection is mainly acquired through inhalation of dehydrated yeast cells from environmental sources, including pigeon excreta and plant debris [3–5]. Within the lungs, this fungus may cause pneumonia, and is able to disseminate to the central nervous system (CNS) where it infects the meninges and brain parenchyma [6–8].

The mortality associated with cryptococcal meningitis varies among different countries and is dependent on several factors, such as the availability and the patient access to antiretroviral therapy (ART), antifungals, as well as the time of diagnosis and elevated CNS opening pressure [9, 10]. In Africa, despite the increasing availability of ART and amphotericin B in some regions, the mortality rate varies from 17 to 62% [11–14]. Despite ART being provided free of charge by the public health service and thus readily available in Brazil, the mortality in the first week of admission is still 42–51%, which is attributed to the advanced immunosuppression at start of HIV treatment and late diagnosis [9, 15]. These rates of mortality are higher than those observed in countries with high GNP (gross national product), such as in Europe (6.5–32%) [10, 16] and North America (15–26%) [17, 18].

The disease is caused by two sibling species subdivided in major molecular types according to different techniques [19–24]: *Cryptococcus neoformans*, with the major molecular types VNI/VNII/VNB (*C*. *neoformans* var. *grubii*, serotype A), VNIV (*C*. *neoformans* var. *neoformans*, serotype D), and VNIII (AD hybrids); and *Cryptococcus gattii* with VGI, VGII, VGIII, and VGIV (serotypes B and C). In addition, some interspecies hybrids such as AB and BD can occur [25–27]. Recently, a new proposal to elevate the major molecular type status to species level was published [28]. However, throughout the current study the classical nomenclature of the *C*. *neoformans* / *C*. *gattii* species complex is used.

Several studies have been performed around the world to identify the major molecular types of the *C*. *neoformans* / *C*. *gattii* species complex from clinical and environmental sources, as well as to develop a better understanding of their molecular epidemiology [29–33]. The lack of a standardized typing technique to compare the results obtained from different countries, lead to standardization and adaptation of a consensus MLST scheme by the working group for genotyping of *C*. *neoformans* and *C*. *gattii* of the International Society for Human and Animal Mycology (ISHAM). This MLST scheme has become an important tool for the characterization of the population genetic structure of the *Cryptococcus* species [20]. Using this MLST scheme, previous studies on *C*. *neoformans* var. *grubii* clearly differentiated VNI/VNII/VNB in addition to the presence of three subpopulations in Asia and up to five subpopulations around the world [34]. Recently, a high genetic diversity of southern African isolates was reported using this MLST scheme [35, 36]. Interestingly, the African continent not only has the distinct VNB genotype, but it was also found to have both VNI and VNII populations more widely distributed [29, 36]. This observation led to the African-origin hypothesis for the evolutionary history of *C*. *neoformans* [29, 37]. A number of epidemiological studies have been performed in Brazil using a range of PCR-based techniques [31, 38, 39]. However, no data on the population structure of *C*. *neoformans* using sequencing-based methods are available. Therefore, it is unknown how the Brazilian isolates fit into a context of the global population structure.

To initiate a better overview of the population genetic structure of *C*. *neoformans*, the major aetiological agent of cryptococcosis in HIV-positive patients in Brazil, the ISHAM consensus MLST scheme was applied to genotype 143 clinical and environmental *C*. *neoformans* isolates from the southeastern Brazilian state Minas Gerais, and the obtained data were then placed in a global context via comparison against the ISHAM-MLST database.

## Methods

### Patients and isolates

From 1999 until 2014, one hundred and forty-three *C*. *neoformans* isolates recovered from clinical and environmental samples were collected in the regional centre for infectious diseases at the teaching hospital of the Triangulo Mineiro Federal University, Uberaba, Minas Gerais state, Brazil (S1 and S2 Tables). Of these, 108 (75%) were clinical isolates, which were recovered from the following body sites: 82 (76%) from cerebrospinal fluid (CSF), 13 (12%) from blood, 11 (10%) from urine, one (1%) from skin, and one (1%) from bronchoalveolar lavage (BAL) fluid. One isolate per patient was selected from most of the cases throughout the study, except in seven situations, where two isolates from different body sites and/or from serial samples were included. For the HIV-positive patients, the CD4^+^ T-cell count and HIV viral load were determined at maximum two weeks after the diagnosis of cryptococcosis for all patients.

The remaining 35 (25%) isolates were recovered from bird droppings obtained in pet shops from different neighbourhoods and debris of trees from surrounding hospital areas (S1 Table).

### *URA5*-RFLP and mating type

Restriction fragment length polymorphism (RFLP) analysis of the orotidine monophosphate pyrophosphorylase (*URA*5) gene was used to confirm the major molecular type of the isolates. Strains WM 148 (serotype A, VNI, AFLP1), WM 626 (serotype A, VNII, AFLP1A and AFLP1B), WM 628 (serotype AD, VNIII, AFLP3), WM 629 (serotype D, VNIV, AFLP2), WM 179 (serotype B, VGI, AFLP4), WM 178 (serotype B, VGII, AFLP6), WM 161 (serotype B, VGIII, AFLP5), and WM 779 (serotype C, VGIV, AFLP7), were used as controls for the identification of the major molecular types of the *C*. *neoformans* / *C*. *gattii* species complexes [40].

The mating type allelic profiles of the *STE20* gene was identified by PCR using the following primers: Aα (JOHE7264/JOHE7266), A**a** (JOHE7270/JOHE7271), Dα (JOHE7267/JOHE7269), and D**a** (JOHE7273/JOHE7274) [41, 42]. To exclude misidentification with AD hybrids homozygous at mating type locus, the *GPA1* gene specific for serotype A (JOHE2596/JOHE3241) and serotype D (JOHE2596/JOHE3240) in addition to the *PAK1* gene specific for serotype A (JOHE3066/JOHE3236) and serotype D (JOHE3066/JOHE3065) were also amplified as previously described [41, 42]. The *C*. *neoformans* strains H99 (Aα, VNI), KN99 (A**a,** VNI), JEC20 (D**a,** VNIV), and JEC21 (Dα, VNIV) were included as controls in all analyses.

### Flow cytometry

Cells were grown in yeast peptone dextrose (YPD) broth overnight with agitation and then washed twice with phosphate buffered saline (PBS). Following washing the cells they were fixed overnight in 70% ethanol at 4°C with mild agitation. Approximately 10^7^ cells were washed with 1 mL NS Buffer (10 mM Tris-HCl pH 7.5, 0.25 M sucrose, 1 mM EDTA, 1 mM MgCl_2_, 0.1 mM CaCl_2_, 0.1 mM ZnCl_2_, 0.55 mM phenylmethylsulfonyl fluoride, 0.049% 2-mercaptoethanol), resuspended in 0.2 mL of NS Buffer containing 14 μL RNase A (Sigma-Aldrich, 1mg/mL) and 14 μL propidium iodide (Sigma-Aldrich, 1mg/mL), and incubated in the dark at room temperature for 4–6 hours. Then, 50 μL of the stained cell mixture was added to 0.5 mL of 1 M Tris pH 7.5 and 1.5 mL PBS [43]. Flow cytometry was performed on 10,000 cells at a slow flow rate with a Becton-Dickinson FACSCanto II. The results were analysed using the software FlowJo (FlowJo, LLC, OR, USA). The haploid reference strains H99 *C*. *neoformans* var. *grubii* VNI and strain CDCR265 *C*. *gattii* VGII and the diploid hybrid strain WM 09.184 *C*. *neoformans* VNII/VNIV were included as controls.

### Multilocus sequence typing (MLST)

The ISHAM consensus MLST scheme for *C*. *neoformans* and *C*. *gattii* was applied in the current study using the amplification conditions previously described [20]. PCR products of the six housekeeping genes *CAP59*, *GPD1*, *LAC1*, *PLB1*, *SOD1*, *URA5*, and the IGS1 region were commercially purified and sequenced by Macrogen Inc., Seoul, South Korea. Sequences were manually edited using the software Sequencher 5.3 (Gene Codes Corporation, Ann Arbor, MI, USA) and aligned using Muscle algorithm available in MEGA 6.06 [44]. The allele types and the sequence types (ST) were identified via sequence comparisons with the *C*. *neoformans* MLST database at http://mlst.mycologylab.org/.

### Nucleotide diversity

The extent of DNA polymorphisms, such as the number of polymorphic sites (S), number of haplotypes (*h*), haplotype diversity (Hd), nucleotide diversity (π) that gives the proportion of nucleotide differences in all haplotypes, average number of nucleotide differences (k), and Watterson’s estimate per sequence (θ_S_) were calculated using DNAsp v5.10.1 available at http://www.ub.edu/dnasp/ [45]. The neutrality tests Tajima’s D, Fu & Li’s D*, Fu & Li’s F*, and Fu’s Fs were also calculated using this program. Negative results in these tests suggest evidence of purifying selection or population size expansion while positive results suggest balancing selection or a decreasing in population size. The nucleotide diversity was calculated in the southeastern Brazilian dataset and the ISHAM-MLST expanded global dataset by including one of each ST per country and excluding the high number of clonal STs from the ISHAM-MLST database, http://mlst.mycologylab.org/. The populations were assigned according to continent of origin.

### Phylogenetic and goeBurst

The phylogenetic analyses and the geographic distribution of *C*. *neoformans* var. *grubii* VNI isolates were performed as follows: First, the best model for the concatenated dataset was chosen from the Bayesian information criterion (BIC) using the software jModelTest 2.1.7 [46, 47]. The Tamura Nei model with invariable sites and gamma distribution (TrNef + I + G) with *p-inv*: 0.955 and alpha shape: 0.832 was selected and used in the phylogenetic analysis. The unrooted maximum likelihood (ML) phylogenetic tree was calculated applying a bootstrap of 1,000 replicates in MEGA v6.06. In addition, the dataset was submitted to the neighbour-joining (NJ) algorithm based on the TrNef + G model [48] to infer the tree congruence. For the ML method, all sites were included in the analyses, while for NJ, all positions containing alignment gaps were eliminated.

To evaluate patterns of evolutionary descent among genotypes according to their source and geographic region, the allelic profile of the southeastern Brazilian and the global dataset were applied to the goeBURST algorithm in PHILOVIZ software available at http://www.phyloviz.net/wiki/ [49]. In this analysis, differences between STs are presented as single locus variant (SLV), double locus variant (DLV), and triple locus variant (TLV) respectively. The concept of a clonal complex (CC) was adopted when a SLV linkage with the founder ST was observed [49, 50].

### Genetic differentiation based on allelic profiles

The genetic differentiation of pre-defined *C*. *neoformans* var. *grubii* VNI populations (e.g. clinical and environmental, and according to the continent of origin) was calculated using the hierarchical Analysis of Molecular Variance (AMOVA) implemented in the software GenoDive v2.0 [51] and Principle Component Analysis (PCA) using the *Adegenet* v2.0.1 package for statistical software R v3.2.4 (https://www.R-project.org/). In addition, the population differentiation test (*F*_*ST*_) from an AMOVA [52, 53], assuming that the isolates were all haploids or homozygous diploids, was used to test the null hypotheses (H_0_) of no population differentiation. Values of *F*_*ST*_ can range from 0, which implies complete panmixis and means that the two populations are interbreeding freely (in these scenario we accept H_0_ and the p value is greater than >0.05), to 1 where all genetic variation is explained by the population structure, and that the two populations do not share any genetic diversity. The *F*_*ST*_ test was calculated with one ST per population (clone-corrected dataset) using 1,000 permutations in the software GenoDive v2.0 and using the *Hierfstat* package in R applying the Mont Carlo test to infer difference among populations in the PCA analysis.

### Linkage disequilibrium and recombination

The presence of recombination within the *C*. *neoformans* var. *grubii* VNI population was inferred using the classical index of association *I*_*A*_ and rBarD. Both indices were calculated using clone corrected allelic profiles in the software Multilocus v1.3 [54] using 1,000 randomizations, which simulate infinite panmixis and compare the values of the observed dataset with those artificially generated by the randomization process. Absence of difference between both datasets (p>0.05) supports the null hypotheses of linkage equilibrium and sexual recombination while significant differences supports linkage disequilibrium (LD) and clonality. The minimal number of recombination events per gene and per population was then calculated in the software DNAsp v. 5.10.1 [45].

The presence of recombination per gene (intragenic), in the concatenated dataset (intergenic) of each population and in the expanded global dataset was also checked by phylogenetic compatibilities of nearby polymorphic sites along single and concatenated sequences in the software SplitsTree v. 4.13.1(http://www.splitstree.org/) [55]. Recombination events were visualized by the formation of parallelograms between neighbours using the reticulated algorithm NeighborNet [55]. This analysis was calculated applying the second best model [Kimura 2-parameter with invariable sites and gamma distribution (K80 + I + G) with *Ti*/*Tv*: 2.895, *p-inv*: 0.957, and alpha shape: 0.920] available in the software jModelTest 2.1.7 due to the absence of the TrNef model in SplitsTree v. 4.13.1 [56]. The Pairwise Homoplasy Index (PHY) test was used to infer if there was statistical significance for recombination.

### Population structure

The actual number of populations (*K*) and their distribution according to source and geographic region was calculated using the Bayesian statistical algorithm [57] implemented in Structure v2.3.4 (http://pritchardlab.stanford.edu/structure.html). The admixture model was selected due to possible presence of migrant individuals among each population. Twenty runs were performed for each value of *K*, ranging from 1 to 10. Each run consisted of Markov Chain Monte Carlo (MCMC) simulations of 1,000,000 interactions and a burn-in period of 100,000 generations [58]. The *K* number was calculated using the software Structure Harvester available at http://taylor0.biology.ucla.edu/structureHarvester/ [59]. The results were graphed using Clumpp and Distruct software’s available at https://web.stanford.edu/group/rosenberglab/software.html [60, 61].

### Coalescence analysis

The coalescence analysis was performed using the Bayesian molecular clock method as implemented in the BEAST v1.8.3 software [62]. The Tamura Nei model with invariable sites and the gamma distribution (TrNef + I + G) model was selected in the software jModelTest 2.1.7 and used in BEAST. In order to find the best-fitting clock model we used the stepping stone sampling marginal likelihood estimator available in the MrBayes v3.2 software [63].

The relaxed lognormal clock was selected to infer the time scale through the incorporation of one internal node calibration in the node separating the VNI and VNB from VNII. The calibration date of 4.5 million years based on genetic distances of different gene sequences was selected from previous studies [64, 65]. A normal prior age distribution with a standard deviation (SD) of 0.25 million years covering the ages of the two previous publications was used in the analysis. Next, the input XML file (S1 Dataset) was generated in the software BEAUTI v1.8.3 with a run of 10^8^ generations, 1 tree sampled per 1,000 generations, and a burn-in of 10% [62]. Two independent runs were performed and the respective files were combined using the LogCombiner v1.8.3 distributed with BEAST (http://beast.bio.ed.ac.uk/) using a burn-in of 10%. The effective sample size (ESS) was higher than 200 in all analyses and was visualized using the software Trace v1.6.0 distributed with BEAST. The tree with the highest log clade credibility was selected in the software TreeAnotator v1.8.3, also distributed with BEAST. The tree presenting the posterior mean and 95% confidence intervals of the time to the most recent common ancestor was visualized using the software FigTree v1.4.3 (http://tree.bio.ed.ac.uk/software/figtree/).

The pattern of ancestry for single genes using the global dataset was also estimated using statistical parsimony, which infers the gene genealogies and the most ancestral haplotype using the software TCS v1.2.1 available at http://darwin.uvigo.es/software/tcs.html [66].

### Statistical analysis

The normality test Shapiro-Wilk was used to infer if the continuous variables presented normal distribution. The Student’s *t* test, calculated using the *car* package for statistical software R (https://www.R-project.org/), was used to analyse differences between two continuous variables. The univariate analysis and odds ratio with 95% confidence interval (CI) was performed with the *epiR* package in R. Those variables that presented a p value < 0.25 in the univariate analysis were included in the multivariate logistic regression analysis calculated using the *stats* package in R.

### Ethics and statement

All samples of the study were retrieved from the culture collection of Mycology Laboratory of the Triangulo Mineiro Federal University. All data were de-identified. Institutional Human Research Ethics approval for the study was obtained from the Research Ethics Board of the Triangulo Mineiro Federal University (protocol #1350).

### Accession numbers

All sequences are deposited in GenBank and their accession numbers are described in S1 Table.

## Results

### Clinical and laboratory analysis

Of the 108 clinical samples studied, only one isolate per patient was included in the 101 cases. In the remaining seven cases, two isolates were included because they were collected from serial CSF samples and/or from different body sites (S2 Table). The underlying clinical information was available for 103 patients, the majority of them (95) had AIDS, three were kidney transplant recipients with or without concurrent diabetes mellitus, three had only diabetes mellitus and/or diabetes mellitus and nephritis, one had systemic lupus erythematosus, and one had Crohn's disease (S2 Table). For statistical analysis, we excluded the duplicate isolates and the patients without the respective information: outcome, CD4^+^ T-cell count, and HIV viral load. Overall, complete clinical information was available for 95 patients, 87 of them HIV-positive. The majority were male (72, 78.9%), with a mean age of 40.17 years [Standard Error (SE) ±11.57]. There was no difference in age between the genders (male = 39.69 ± SE 11.29 vs. female = 41.65 ± SE 12.06, p = 0.48). Among the patients whose isolates were included, 56 (58.9%) had fatal outcome, regardless of gender (p = 0.238), and 64.9% (55/87) when only the HIV-infected population was considered. HIV infection was strongly correlated with death in both univariate and multivariate analyses and presented an odds ratio of 14.84 times when compared with non HIV-infected patients (Table 1). Within the HIV-infected group with CD4^+^ T-cell count and HIV viral load information available (77 patients), the CD4^+^ T-cell counts revealed strong association with immunosuppression as half of the patients had counts below 50 mm^3^/ml. On the other hand, neither CD4^+^ T-cell count nor HIV viral load showed a statistical association with fatality (Table 1).

**Table 1:** Characteristics associated with deaths of the Brazilian patients.

Variable	% Deaths (n/total)	Univariate		Multivariate	
		p-value	OR (95% CI)	p-value	OR (95% CI)
**Gender**		0.238	0.54 (0.20–1.49)	0.132	0.42 (0.14–1.29)
Male	42.1 (40/95)				
Female	16.8 (16/95)				
**ST93**		0.901	0.94 (0.37–2.39)		
Yes	43.1 (41/95)				
No	15.7 (15/95)				
**Cluster**		0.595	1.34 (0.45–4.00)		
Minor	47.3 (45/95)				
Major	11.5 (11/95)				
**HIV**		**0.023**	12.03 (1.41–102.27)	**0.017**	14.84 (1.64–133.63)
Positive	57.9 (55/95)				
Negative	1.05 (1/95)				
**CD4**^**+**^ **T-cell**^#^		0.086	2.34 (0.88–6.22)		
<50 cells/mm^3^	29.8 (23/77)				
>50 cells/mm^3^	35.0 (27/77)				
**HIV load**^#^		0.928	0.95 (0.36–2.52)		
<30.000 copies/ml	45.5 (32/77)				
>30.000 copies/ml	23.3 (18/77)				

^#^: CD4^+^ T-cell counts and HIV viral load were only calculated for HIV patients and thus not included in the multivariate analysis.

P values < 0.05 are highlighted in bold

### Molecular type, mating type, serotype, MLST, and nucleotide diversity

**Fig 1:**
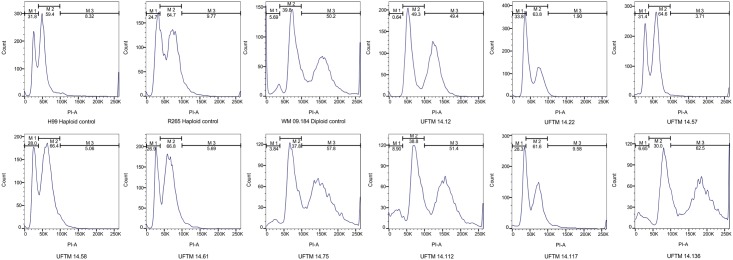
Representative flow cytometry curves of selected southeastern Brazilian *Cryptococcus neoformans* var. *grubii* VNI isolates. Out of 143 isolates, 12 were identified as diploid. The haploid strains H99 *C*. *neoformans* var. *grubii* VNI and CDCR265 *C*. *gattii* VGII and the diploid hybrid strain WM 09.184 *C*. *neoformans* VNII/VNIV were included in each flow run as controls.

*URA5*-RFLP analysis identified all isolates as *C*. *neoformans* var. *grubii* major molecular type VNI. They were all mating type alpha and serotype A based on PCR with primer sets specific for the mating type locus *STE20*, as well as the *GPA1*, and *PAK1* genes. The majority of the isolates were haploid, but 10 (9.2%) of the clinical and two (5.7%) of the environmental isolates were diploid (double DNA content) based on flow cytometry (Fig 1). Taken together, these results show a high prevalence (n = 131; 91.6%) of haploid, serotype A/mating type alpha (Aα) isolates and a minority (n = 12; 8.4%) of diploid (AαAα) isolates (S1 Table).

MLST analysis of the 143 southeastern Brazilian isolates demonstrated the presence of 13 sequences types (ST). The majority of them represented “high frequency” STs, such as ST93 (91; 63.6%), ST77 (19; 13.3%), ST23 (7; 4.9%), and ST63 (6; 4.2%). The remaining STs were represented by one to three isolates, and ST540 was a newly described sequence type. Ten of the 13 STs were identified in clinical isolates, while seven were present in environmental isolates. ST23, ST32, ST39, ST63, ST289, and ST540 were only found among clinical strains, while ST2, ST15, and ST77 were found only in environmental isolates. ST93 represented 10 clinical homozygous diploid isolates while ST77 represented the two environmental strains. Of the seven patients from whom two samples each were studied, one patient had isolates (UFTM 14.61/UFTM 14.75) of the same ST (e.g. ST93), but different ploidy (e.g. haploid and diploid). Two other patients were co-infected by two isolates presenting different STs with different ploidy (UFTM 14.112, ST93 diploid/UFTM 14.117, ST23 haploid, and UFTM 14.96, ST32 haploid/UFTM 14.114, ST93 diploid) (Fig 1, S1 and S2 Tables). The remaining patients were co-infected by the same STs, all of them haploids.

To place the southeastern Brazilian isolates in a global context, we expanded the analysis by including one of each of the STs per country, excluding the high number of isolates from clonal STs from the ISHAM-MLST database (http://mlst.mycologylab.org/). It is important to mention that human migration will have a blurring effect on the selection of the ST per country, especially because cryptococcal cells can be dormant for decades in the host’s body. To overcome this fact, an effort was made to select most STs from references that didn’t mention migration, or that most of the isolates were from autochthonous cases. A total of 179 isolates and 91 different STs were included, with 148 clinical, 23 environmental, three veterinary, and five isolates from unknown sources (S1 Table). The majority (150; 83.8%) belonged to mating type alpha, four (2.2%) to mating type **a**, and the mating type information for the remaining isolates was not available. Serotype A information was available for 44 isolates (S1 Table).

The nucleotide sequences of the southeastern Brazilian *C*. *neoformans* var. *grubii* VNI isolates showed between 0 to 13 polymorphic sites (Table 2). The IGS1 region presented the highest nucleotide diversity (π = 0.0045) and mutation rate (θ_S_ = 2.348), followed by *GPD1* (π = 0.0012; θ_S_ = 1.084). In contrast, *SOD1* was the least variable genetic locus, with only one allele type. The average estimates of these statistics for the concatenated sequences were also low (Hd = 0.507, π  = 0.0012, and θ_S_ = 4.877), reflecting the low genetic diversity of the isolates. The neutrality tests Tajima’s D, Fu and Li’s D*, Fu and Li’s F*, and Fu’s F_S_ showed evidence of balancing selection or expansion of rare polymorphisms for all loci in the southeastern Brazilian population (Table 2). Using the expanded global dataset and distributing the isolates into subpopulations, African populations presented the highest genetic diversity (h = 43, π  = 0.0027, and θ_S_ = 13.88 for the concatenated sequences). The lowest genetic diversity was observed among South American populations (h = 13, π  = 0.0012), followed by North American (h = 22, π  = 0.0020), Asian (h = 37, π  = 0.0020), and European populations (h = 29, π  = 0.0022). Overall, the IGS1 locus was the most variable region (π  = 0.0074), followed by *SOD1* (π  = 0.0053), and *LAC1* (π  = 0.0021). The neutrality tests for the overall *C*. *neoformans* var. *grubii* VNI population showed evidence of purifying selection or population expansion for all loci (Table 2).

**Table 2:** Polymorphism data analyses of the single loci and the concatenated dataset of the different *Cryptococcus neoformans* var. *grubii* VNI continental populations.

Population	Locus	Length	S	*h*	*Hd*	π	*k*	θ_S_	*D*	*F*_*D*_	*F*_*F*_	*F*_*S*_
**Africa** **(n = 61)**	*CAP59*	560	6	3	0.432	0.0010	0.573	1.282	-1.355	**-3.287**	**-3.132**	0.791
	*GPD1*	544	9	9	0.555	0.0022	1.225	1.923	-0.980	-1.405	-1.491	-2.827
	IGS1	724	14	7	0.615	0.0069	5.043	2.992	1.702	0.609	1.173	5.408
	*LAC1*	471	10	6	0.651	0.0029	1.402	2.137	-0.949	-1.812	-1.799	0.206
	*PLB1*	534	6	6	0.732	0.0021	1.173	1.282	0.208	0.269	0.136	-0.348
	*SOD1*	537	17	9	0.247	0.0015	0.811	3.6.33	**-2.351**	-2.349	**-2.790**	-4.825
	*URA5*	637	3	4	0.591	0.0010	0.691	0.641	0.153	0.871	0.760	0.074
	*Concatenated*	4,007	65	43	0.985	0.0027	10.91	13.88	-0.771	-1.717	-1.622	-21.43
**Asia** **(n = 69)**	*CAP59*	560	1	2	0.251	0.0004	0.251	0.208	0.258	0.517	0.512	0.787
	*GPD1*	544	5	5	0.522	0.0014	0.775	1.041	-0.580	1.068	0.635	-0.536
	IGS1	724	13	5	0.497	0.0065	4.737	2.706	2.136*	0.962	1.625	8.313
	*LAC1*	473	13	7	0.686	0.0024	1.141	2.706	-1.646	**-2.297**	**-2.921**	-1.144
	*PLB1*	533	4	4	0.483	0.0013	0.727	0.833	-0.268	0.973	0.683	0.290
	*SOD1*	536	1	2	0.057	0.0001	0.057	0.208	-0.901	0.517	0.116	-1.164
	*URA5*	637	4	5	0.533	0.0009	0.593	0.833	-0.610	-1.331	-1.293	-1.264
	*Concatenated*	4,007	41	37	0.970	0.0020	8.282	8.534	-0.096	-0.542	-0.445	-14.69
**Europe** **(n = 39)**	*CAP59*	560	9	5	0.586	0.0017	0.964	2.129	-1.607	**-2.998**	**-3.002**	-0.455
	*GPD1*	544	4	3	0.448	0.0014	0.722	0.946	-0.442	1.029	0.684	1.188
	IGS1	725	14	5	0.366	0.0032	2.348	3.311	-0.922	1.082	0.505	2.320
	*LAC1*	471	12	7	0.791	0.0039	1.852	2.838	-1.075	**-2.575**	-2.461	-0.283
	*PLB1*	533	4	4	0.704	0.0028	1.530	0.946	1.483	1.029	1.357	1.893
	*SOD1*	537	3	3	0.101	0.0002	0.154	0.710	-1.718	**-2.865**	**-2.935**	-1.962
	*URA5*	637	12	5	0.568	0.0018	1.196	2.838	-1.791	-2.069	-2.329	0.132
	*Concatenated*	4,007	58	29	0.984	0.0022	8.815	13.71	-1.287	-1.635	-1.798	-13.31
**SouthAmerica**^#^ **(n = 144)**	*CAP59*	569	1	2	0.222	0.0004	0.222	0.181	0.2431	0.471	0.470	0.828
	*GPD1*	544	6	5	0.502	0.0012	0.695	1.084	-0.761	1.091	0.548	-0.305
	IGS1	724	13	5	0.501	0.0045	3.242	2.348	0.980	1.497	1.562	7.108
	*LAC1*	471	4	4	0.232	0.0007	0.348	0.722	-0.969	0.910	0.348	-1.062
	*PLB1*	533	2	3	0.458	0.0009	0.497	0.361	0.544	0.658	0.731	0.935
	*SOD1*	536	0	1	0.000	0.0000	0.000	0.000	na	na	na	na
	*URA5*	637	1	2	0.119	0.0001	0.119	0.181	-0.366	0.471	0.252	-0.095
	*Concatenated*	4,014	27	13	0.507	0.0012	5.123	4.877	0.146	**1.922**	1.455	2.724
**NorthAmerica** **(n = 9)**	*CAP59*	560	1	2	0.556	0.0006	0.556	0.368	1.401	0.840	1.069	1.015
	*GPD1*	544	2	3	0.417	0.0008	0.444	0.736	-1.362	-1.505	-1.626	-1.081
	IGS1	724	11	4	0.583	0.0063	4.556	4.047	0.590	1.162	1.145	2.353
	*LAC1*	471	4	4	0.750	0.0033	1.556	1.472	0.231	-0.264	-0.162	-0.133
	*PLB1*	533	2	3	0.667	0.0017	0.944	0.736	0.975	1.063	1.151	0.245
	*SOD1*	536	1	2	0.222	0.0004	0.222	0.368	-1.088	-1.189	-1.282	-0.263
	*URA5*	637	1	2	0.500	0.0007	0.500	0.368	0.986	0.840	0.962	0.849
	*Concatenated*	4,005	22	9	1.000	0.0020	8.778	8.095	0.417	0.537	0.570	-3.146
**Overall** **(n = 322)**	*CAP59*	569	9	5	0.350	0.0007	0.417	1.417	-1.518	-1.372	-1.713	-1.256
	*GPD1*	544	10	11	0.628	0.0018	0.987	1.575	-0.824	-1.984	-1.872	-3.344
	IGS1	725	18	12	0.628	0.0074	5.371	2.283	2.014	0.566	1.386	6.430
	*LAC1*	473	17	9	0.569	0.0021	1.024	2.677	-1.533	-1.399	-1.753	-1.566
	*PLB1*	534	6	6	0.664	0.0017	0.933	0.945	-0.024	-0.056	-0.054	0.304
	*SOD1*	537	18	10	0.079	0.0053	0.192	2.835	**-2.339**	**-3.013**	**-3.315**	-13.30
	*URA5*	637	15	8	0.415	0.0008	0.525	2.362	**-1.884**	**-4.314**	**-4.066**	-3.570
	*Concatenated*	4,019	93	86	0.895	0.0023	9.449	14.64	-1.077	**-3.254**	**-2.629**	-34.15

^**#**^: South American population represents all isolates from Brazil and one from Argentina

**S**: Number of polymorphic sites

***h***: Haplotype number

***Hd***: Haplotype diversity

**π**: Nucleotide diversity

***k***: Average number of nucleotide per sequence

P values < 0.05 are highlighted in bold.

### Genetic variation, phylogeny, and geographic distribution

**Fig 2:**
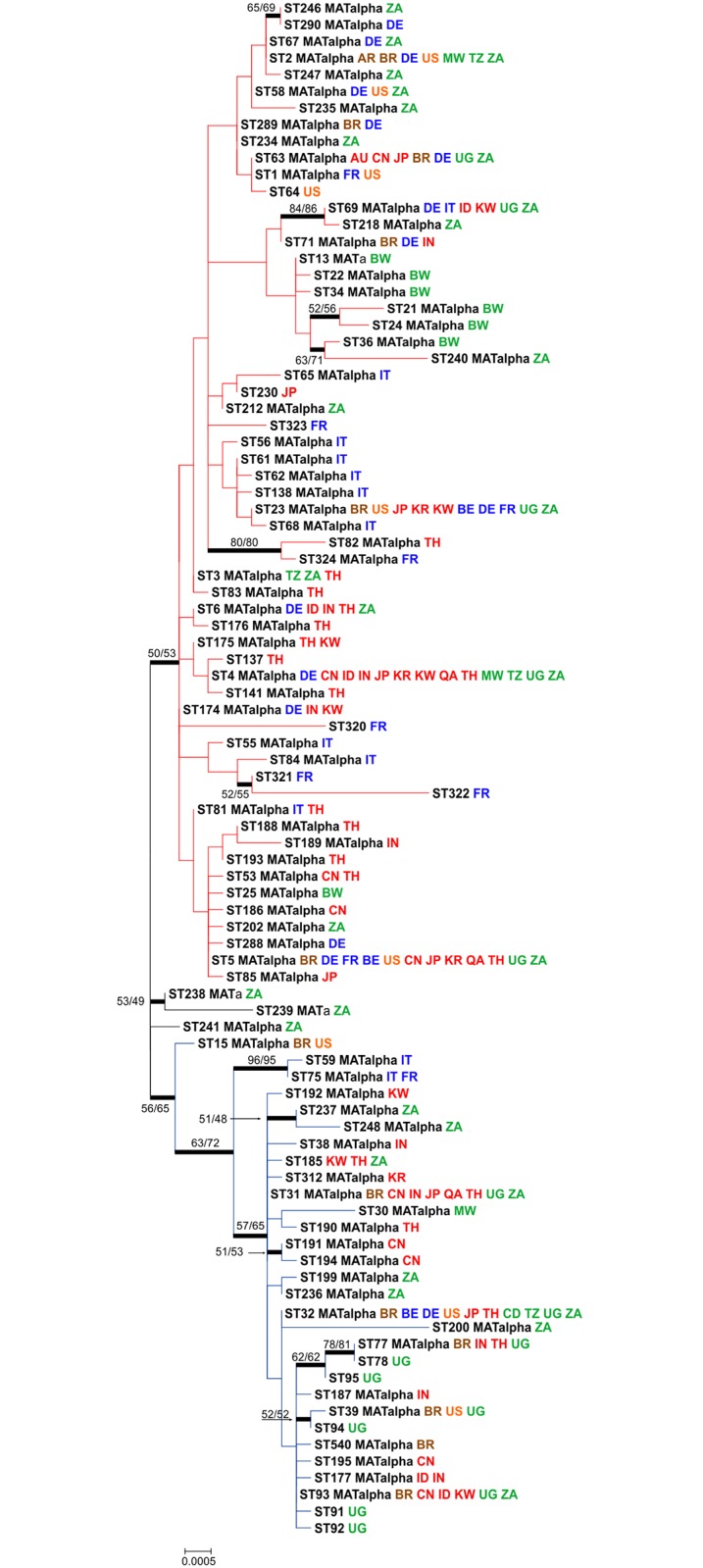
Maximum likelihood (ML) consensus tree of the global *Cryptococcus neoformans* var. *grubii* VNI dataset using the concatenated sequences of the seven MLST loci (*CAP59*, *GPD1*, *LAC1*, *PLB1*, *SOD1*, *URA5*, and the IGS1 region). The tree with the highest log likelihood (-7,114) drawn to scale with branch lengths measuring the number of substitutions per site is shown. Bootstrap values >50% based on 1,000 replicates for the ML and the neighbour-joining (NJ) analyses, which showed similar topologies, are presented close to the branches. The analysis involved 92 nucleotide sequences with 4,010 positions revealing the two main clusters (red = major and blue = minor). The isolates are described according to the sequence type number (ST), followed by mating type (**a** or α) and country of isolation, which are abbreviated according to the alfa-2 code of ISO 3166–1. The colours of each country represent the continent of origin as follows: blue: Europe, brown: South America, green: Africa, orange: North America, red: Asia.

The maximum likelihood analysis of the global dataset revealed the presence of two major clusters within the *C*. *neoformans* var. *grubii* VNI population (Fig 2; S1 Fig). The first (minor) cluster was composed of 30 different STs and had higher bootstrap support than the second (major) cluster with 59 STs. Three STs (e.g. ST238, ST239, and ST241) were grouped between the two major groups in the phylogenetic analysis. Both clusters were composed of isolates recovered from five continents, although isolates recovered from Europe and Asia were mainly found within the major cluster. All but the newly identified ST540 from Brazil had been previously reported from different regions of the world (Fig 2).

**Fig 3:**
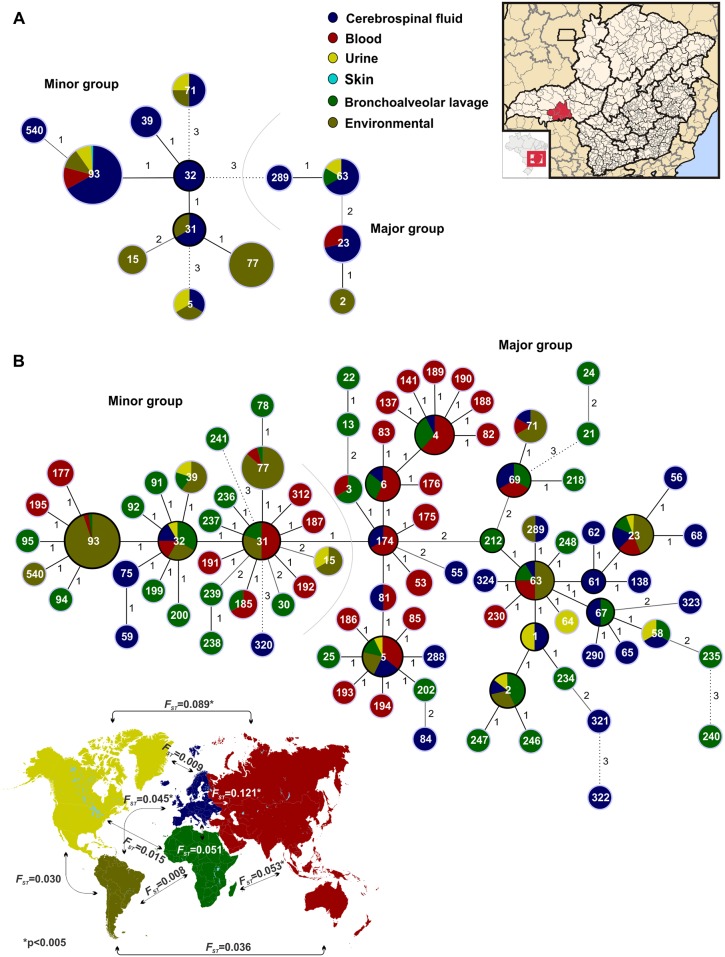
**A) Minimum spanning tree showing the distribution of the 143 *Cryptococcus neoformans* var. *grubii* VNI isolates from southeastern Brazil according to the source of isolation**. Two main clusters were identified. The first composed of the clonal complexes (CC) CC31 and CC32, which are both linked with the most frequent STs found in the study, ST93 (recovered from both clinical and environmental samples) and ST77 (recovered only from environmental samples). The second main cluster is represented by the ST289, ST63, ST23, and ST2. Each circle represents a unique ST, and the circumference is proportional to the number of isolates within each ST. Clonal complexes surrounded by black circles represent the ancestors in the network. Solid, grey and dashed branches represent at least one, two, and three differences in alleles, respectively. **B) Minimum spanning tree of the isolates presented in A, and the inclusion of 181 STs identified in different continents, retrieved from the ISHAM-MLST database (**http://mlst.mycologylab.org/**).** Fifteen CCs were identified, with the CC174 presenting a central role in the network. This picture shows the pattern of clonality of the VNI genotype, the widely distributed STs (e.g. ST5, ST23, ST32, ST63), the two main clusters, and the broad distribution of African isolates, highlighted in green. The STs are described according to the colours of the map as follows: blue: Europe, brown: South America, green: Africa, yellow: North America, red: Asia. The map also displays the pairwise *F*_*ST*_ tests calculated by AMOVA, with African, North American, and South American populations presenting similar populations, whereas the Asian and European populations are more differentiated.

The goeBurst analysis was applied to infer patterns of evolutionary descent among clusters of related genotypes and to identify groups within populations in the southeastern Brazilian as well as in the global datasets (Fig 3). The goeBurst analysis differentiated the southeastern Brazilian isolates into two main clusters and two more distant STs, ST71 and ST5 (Fig 3A). The clonal complex (CC) CC32 and its descendants ST39, ST93, and ST31, in addition to an additional three linked STs (e.g. ST540, ST15, and ST77) were clustered together. These seven STs were separated by three alleles [e.g. triple locus variant (TLV)] from the second group, which was composed of ST289, ST63, ST23, and ST2. The results did not show any pattern of differentiation based on their source (e.g. clinical or environmental), although the group that contained ST63 and its descendants was mainly represented among clinical isolates (Fig 3A).

Expanding this analysis to the global dataset that contained isolates differentiated by continents, the two main clusters of the southeastern Brazilian isolates were also separated (Fig 3B). The first major cluster composed of CC174 and its single locus variant (SLV) descendants ST3, ST6, ST175, ST53, and ST81 and another 10 CCs (e.g. CC6, CC4, CC5, CC212, CC69, CC63, CC1, CC2, CC67, CC61, and CC23) is presented in a star-like shape, indicating the clonal distribution of *C*. *neoformans* var. *grubii* VNI. This major cluster is separated from the second minor cluster due to a difference in one allele (e.g. SLV). The minor cluster is composed of CC31, CC32, CC93 and its descendants (Fig 3B). Five STs (ST241, ST320, ST322, ST240, and ST21) presented a difference in three alleles (TLV) in the overall minimum-spanning tree generated by goeBurst. There was no visual clustering among geographic distribution and population groups found in the goeBurst analysis, although isolates from Europe were more frequently clustered in the major group.

**Fig 4:**
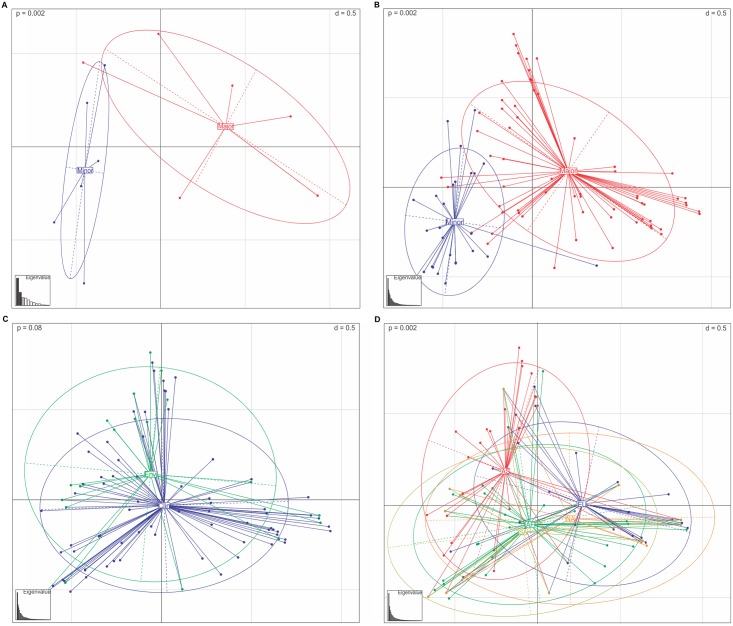
Principle Components Analysis of *Cryptococcus neoformans* var. *grubii* VNI allelic profiles depicted according to: A) the southeastern Brazilian population (13 STs), B) *C*. *neoformans* population using the global dataset (92 STs), C) isolates distributed according to clinical and environmental sources (104 STs), and D) distributed according to continents (136 STs). Dots represent STs linked by coloured lines to form clusters, proportional in sizes to the number of isolates. The two main clusters identified in the *C*. *neoformans* population are highlighted in blue (minor) and red (major). The samples recovered from clinical (Clin) and environmental (Env) sources are highlighted in blue and green respectively. The colours of each continent of origin are described as follows: blue: Europe (EU), brown: South America (SA), green: Africa (AF), orange: North America (NA), red: Asia (AS). Eigenvalues highlighting the two first components are represented in the bar plot. The p-value calculated using the Mont Carlo test is described in the upper left side.

To better understand the distribution of the genetic diversity, Principle Component Analysis (PCA) and hierarchical Analysis of Molecular Variance (AMOVA) were performed. The PCA showed that the southeastern Brazilian and global *C*. *neoformans* var. *grubii* datasets differentiate two major clusters previously identified in the goeBurst analysis (Fig 4A and 4B). Using the global dataset, no support for differences between the clinical and environmental populations was found using PCA (Fig 4C). AMOVA showed that the majority of variance components were found distributed within, rather than between populations (Table 3). In contrast, when dividing the populations according to continents, statistical support (p = 0.002) was found that accounted for differences between populations (Table 3 and Fig 4D). The pairwise *F*_*ST*_ tests were calculated among populations, and showed that Asia and Europe had distinct subpopulations, while Africa, North America, and South America did not have statistical support for differentiation (see map of Fig 3B).

**Table 3:** Hierarchical Analysis of Molecular Variance (AMOVA) of different continental populations of *Cryptococcus neoformans* var. *grubii* VNI.

	d.f	Sum of squares	Variance components (%)	*F*_*ST*_
**All isolates: Environmental (19) and Clinical (85)**				
**Between population**	1	3.843	0.000	0.001
**Within population**	102	402.15	1.971 (100)	
**All isolates: AF (35), AS (37), EU (32), NA (9), SA (13)**				
**Between population**	4	33.646	0.093 (4.9)	**0.049**
**Within population**	131	479.41	1.830 (95.1)	

**AF**: Africa

**AS**: Asia

**EU**: Europe

**NA**: North America

**SA**: South America

**d.f**: Degrees of freedom

P value < 0.005 is highlighted in bold

### Recombination and linkage disequilibrium

The index of association (*I*_*A*_) and rBarD values of the different *C*. *neoformans* var. *grubii* VNI subpopulations were calculated using a clone corrected dataset in order to avoid bias of “high frequency” sequence types in the analysis. Both results strongly reject (p<0.005) the null hypothesis of linkage equilibrium and free recombination in the African, European, and South American subpopulations, while this hypothesis was not rejected for the Asian and North American populations (Table 4). Next, we checked the presence of recombination using the PHI test and the minimal number of recombination events per gene was calculated within each population group and per gene in the global dataset. The PHI test did not show evidence of recombination for any of the populations studied using the single gene datasets. However, all but the North America population showed evidence for recombination using the concatenated dataset (p value of PHI test: Africa: 0.000004, Asia: 0.004, Europe: 0.001, North America: 0.107, South America: 0.013). In addition, one recombination event could be identified in the *SOD1* gene in the African population and one in the *GPD1* gene of the Asia population (Table 4). Taken together, the results from the global dataset showed that the European and South American isolates reproduced clonally. The Asian population showed linkage equilibrium and one event of recombination for the *GPD1* gene while the African isolates present mainly a clonal reproduction with the highest number of recombination events that were uncovered in our dataset. The low numbers of STs available in the database from North America limited a detailed interpretation of these results.

**Table 4:** Multilocus linkage disequilibrium and recombination analyses amongst *Cryptococcus neoformans* var. *grubii* VNI continental populations.

Population^#^	*I*_*A*_	p-value	rBarD	p-value	*Rm* per gene
**Africa**					
*n* = 45	0.287	**0.002**	0.048	**0.002**	*SOD1* = 1
**Asia**					
*n* = 37	0.024	0.378	0.004	0.378	*GPD1* = 1
**Europe**					
*n =* 32	0.296	**0.010**	0.010	**0.001**	0
**North America**					
*n =* 9	0.328	0.111	0.055	0.111	0
**South America**					
*n =* 13	0.359	**0.038**	0.072	**0.038**	0
**Overall**					
*n =* 93	0.233	**0.001**	0.039	**0.001**	*GPD1* = 1; IGS1 = 2; *SOD1 = 1*

^**#**^: Calculated using the clone corrected dataset

***I***_***A***_: Index of association

**rBarD**: Modified statistic of *I*_*A*_

***Rm***: Minimal number of recombination

P values < 0.05 are highlighted in bold

When the global *C*. *neoformans* var. *grubii* VNI population was analysed together, no significant evidence of linkage equilibrium was observed, and the intragenic PHI test showed no statistical significance for recombination events per gene, even though a few recombination events in the *SOD1* and *GPD1* genes in addition to the IGS1 region were detected (Table 4). In the concatenated (intergenic) dataset the PHI test showed evidence (PHI, p<0.0001) of recombination (S2 Fig). The two major clusters identified in the previous analyses were again differentiated by this analysis (S2 Fig).

### Population structure and coalescence

**Fig 5:**
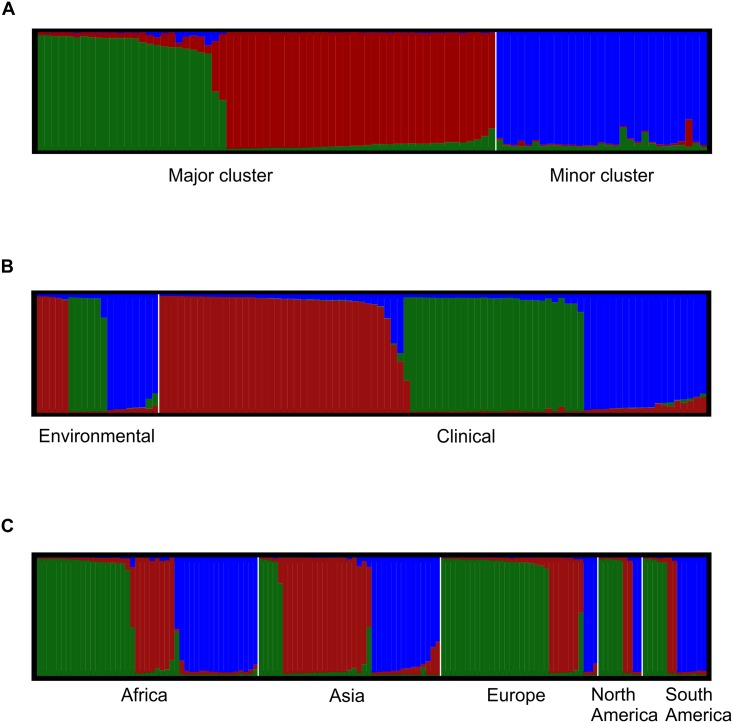
Structure analyses of *Cryptococcus neoformans* var. *grubii* VNI showing that *K* = 3 is the actual number of subpopulations. Clusters of individuals based on prior-defined populations according to: A) major and minor main clusters identified in the phylogenetic and coalescence analyses using the expanded global dataset (92 isolates), B) clinical and environmental isolates (104 isolates), and C) according to continents (136 isolates). Each vertical line represents one ST and the colours represent the most likely ancestry of each individual from the population. Individuals with multiple colours have admixed genotypes from the prior-defined subpopulations. One ST per region and/or per pre-defined subpopulation was used throughout the whole analyses.

Structure software was used to identify the number of populations (*K*) in our dataset and to check for the presence of migrant individuals. We first analysed the global dataset and the results showed three subpopulations with the highest explanatory power. Structure could differentiate both clusters (e.g. major and minor) in addition to one more subpopulation within the major cluster (Fig 5A, S3A Fig). Using the southeastern Brazilian and global datasets, pre-defining the clinical and environmental populations, we also observed three subpopulations, without any differentiation (*F*_*ST*_ test, p = 0.086) between them (Fig 5B, S3B Fig). Furthermore, despite three subpopulations being identified when the isolates were divided by continents (Fig 5C, S3C Fig), some continents were composed of more individuals belonging to one (e.g. Asia, mainly represented by one subpopulation, coloured in red) or another (e.g. Europe, represented mainly by isolates coloured in green) population. The remaining three continents contained isolates from all three subpopulations. Taken together, these data support that there are two main clusters with three subpopulations in *C*. *neoformans* var. *grubii* VNI, which can be found in both clinical and environmental isolates. Recombination events in some isolates could be observed as mosaics of multiple small chromosomal chunks depicted in the Structure analyses (Fig 5).

**Fig 6:**
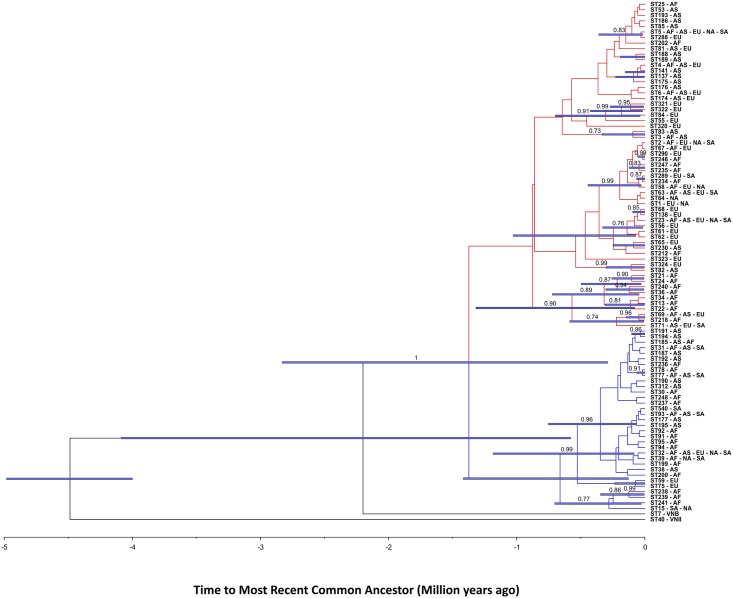
Coalescence gene genealogy of the *Cryptococcus neoformans* var. *grubii* VNI isolates evidencing the presence of two main clusters that separated 0.29 to 2.8 million years ago. Blue bars represent the 95% high posterior probability of the ages in the branches with a posterior probability limit of 0.5. The Bayesian posterior support values higher than 70% are described in the branches of the tree. One ST representing the VNB (ST7) and one representing the VNII (ST40) lineage were included in the analysis as out-groups. The continents of origin of the isolates are abbreviated as follows: AS: Asia, AF: Africa, EU: Europe, SA: South America, NA: North America.

Finally, coalescence analysis also clearly differentiated both clusters and showed that VNI isolates diverged from VNB (ST7) around 0.58 to 4.0 million years ago [effective sample size (ESS) = 5,733] according to the best representative sample of the model used (S1 Dataset). The time to the most recent common ancestor (TMRCA) of the two main VNI clusters was 0.29 to 2.8 million years ago (ESS = 1,604) (Fig 6). The ancestral pattern based on single genes was calculated and showed that, for the majority of the MLST genes, the African alleles are situated on the top, as well as in a terminal position, of the network inferring its descendant profile (S4 Fig).

## Discussion

*Cryptococcus neoformans* is by far the most common pathogen causing meningitis in HIV-infected patients around the world. In Brazil, there are around 734,000 (610,000–1,000,000) estimated people living with HIV and 16,000 (9,900 to 23,000) deaths just in 2014 according to UNAIDS (http://unaids.org.br/). Amongst developing countries, Brazil was one of the first to offer free of charge ART in 1996 through the public health system, which has contributed to the dramatic decline in mortality caused by AIDS (http://unaids.org.br/). The mortality attributed to cryptococcal meningitis has also decreased from as high as 90% before the ART era to 30–55% mortality at 10-week after its introduction [9, 67]. Despite the broad free availability of ART, these numbers still remain high (up to 60% in our study) compared to countries with high GNP (6,5–32%) [10, 16–18] and require more investment from the public health system. Our data also reinforce the current clinical picture of cryptococcal meningitis in Brazil, which is mainly represented by adult males, in the median age of life, with late HIV diagnosis, severe immunosuppression, and disseminated fungal disease at admission for HIV treatment. This late HIV diagnosis is in contrast to freely available HIV diagnostic tests throughout the public health system in Brazil. This observation reflects the stigma that HIV infection has in the Brazilian society and represents one of the main challenges for public health policy-makers in Brazil. On the other hand, despite *C*. *gattii* infection has been described in children [68], our results also confirm the rarity of cryptococcosis due to *C*. *neoformans* in pediatric cases [69, 70] with the youngest patient presenting at age 18.

Few studies on *C*. *neoformans* in Brazil and other South American countries have linked clinical data with molecular epidemiological data. Furthermore, most of the studies to date conducted to better understand the genetic population structure of *C*. *neoformans* in these countries have only been performed using a range of PCR-based techniques using anonymous genetic markers [39, 40, 71–74]. As a consequence, little information about molecular epidemiology of this pathogen has been generated using sequencing-based methods. These methods aid in the integration of results into global databases, providing insights into the evolutionary ecology and molecular epidemiology of the pathogen. Since 2009, the ISHAM consensus MLST scheme has been used in several parts of the world, especially in regions with high prevalence of HIV-infected patients, such as Southeast Asia [34, 75, 76] and Africa [35, 36, 77], but also in different countries across Europe [10, 78], and North America [79]. Our results show a high prevalence of ST93 (91/143, 63.6%) for both clinical and environmental isolates and ST77 in half (19/35, 54.2%) of the environmental isolates (S1 Table). ST93 has been mainly recovered from HIV-positive patients in several countries, but is more prevalent in Indonesia, India, and Uganda [34, 77, 80]. ST77 was mostly recovered from clinical samples in India, but also in few patients from France, Thailand, and Uganda [34, 77, 80]. The presence of both of these STs in clinical and environmental samples, in addition to the absence of the genetic differences between these two populations suggests that STs have not adapted specifically to environmental or clinical sources (see, e.g., Figs 3A, 4C and 5B).

Interestingly, some of these “high frequency” STs were diploid, some of them co-infecting patients with haploid strains (S2 Table). To rule out any misinterpretations of these results due to contamination with different colonies, two independent flow runs were performed using single colonies for all diploid isolates. In the *C*. *neoformans / C*. *gattii* species complex, diploid AD hybrids, which are the result of matting between *C*. *neoformans* strains of serotypes A and D [81], occur more frequent than hybrids, which are the result of matting between the two sibling species *C*. *neoformans* and *C*. *gattii* [19, 25–27]. Homozygous diploids (AαAα or A**a**A**a**) have also been found in clinical specimens from South Africa [82] where the percentage (9%) is similar to this study (9.8%). Although, herein all isolates presented the diploid profile AαAα, as analysed by flow cytometry, and PCR screening for the mating type specific *STE20* gene and serotype specific *GPD1* and *PAK1* genes. In the southeastern Brazilian region, dual infections were previously described in two AIDS patients, one by two RAPD subtypes of *C*. *neoformans* var. *grubii* VNI genotype, and the other by two isolates each from *C*. *neoformans* var. *grubii* VNI and *C*. *gattii* VGII in a reference center of Rio de Janeiro [83]. A previous study with 500 isolates from different geographic regions also showed that 8% of the isolates were diploid [84]. Two mechanisms have been proposed to explain the presence of diploid isolates in a population where the majority of the isolates are haploid. First, intra-varietal diploidization produced by fusion of two genetically distinct alpha cells. Second, autodiploidization generated when identical copies of the genome arise via either endoreplication or clonal mating [84, 85]. Although we did not detect any mating type **a** among our isolates, we cannot rule out the possibility of its presence in the environment, which would keep the normal mating cycle of *C*. *neoformans* (e.g. α-**a** sexual reproduction) going. However, autodiploidization seems to be an alternative possibility, as has been previously suggested [84, 86]. The presence of both haploids and diploids in a population and/or a shift in the ploidy has also been shown to increase fitness during stress conditions such as growth in the presence of different concentrations of azoles, nitrosative and oxidative stress [87, 88]. This mechanism may be used by tetraploid titan cells, which play an important role in establishment of the pulmonary infection and subsequent dissemination to the CNS [87, 89].

Surprisingly, all but one of the MLST STs described here were also present in different regions of the world. This result is highlighted by the goeBurst analysis, where all isolates from southeastern Brazil occur either in the same clonal complex or form a close network with STs from other countries and continents (Fig 3B). However, differences in prevalence are also important to highlight. For example, ST5, which was one of the most prevalent STs amongst the clinical isolates from Europe and Asia [34, 90–92], was only identified in two clinical and one environmental isolate from Brazil. Other STs seem to be very uncommon, for instance, ST289 was only found in one clinical sample in this study and in few occasions in Germany [90]. Since some STs are more prevalent in different regions, and to develop an overarching view of the *C*. *neoformans* var. *grubii* VNI global population, we separated the entire MLST dataset according to continents. This analysis clearly showed that the highest genetic diversity was observed in the African population and the lowest in the South American population (Table 2). The African isolates exhibited the greatest number of polymorphic sites, haplotypes, nucleotide diversity, as well as mutation rate. The high genetic diversity within the African population has already been previously described [36, 37, 75, 93], however in these studies, all three molecular types of *C*. *neoformans* var. *grubii* serotype A (e.g. VNI, VNB, and VNII) and only few or no isolates from South America were included. These results highlight that the high variability is not just only due to the African VNB genotype, but also when only the VNI subpopulation were considered.

The low variability observed amongst South American isolates studied here could be due to the limited geographical scale of our study. However, as the first sequence based typing study of *C*. *neoformans* var. *grubii* VNI in South America, the current study was conducted on a wide variety of clinical and environmental isolates obtained in an infectious diseases centre of a university teaching hospital which receives patients from 27 cities in Southeast Brazil, increasing the variability sampled. To verify the suggested finding of a mainly clonal *C*. *neoformans* var. *grubii* VNI population responsible for the high mortality in HIV-positive patients in Brazil, a larger study including isolates from different regions of the country and from different countries in South America would be desirable. Brazil lacks systematic epidemiological studies on cryptococcosis and the diagnosis is markedly delayed. To overcome these obstacles, 32 multidisciplinary research groups from a variety of universities and medical centers in Brazil set up the Brazilian Cryptococcosis Network (RCB) in 2013, which aims to study the epidemiological markers and clinical outcome of primary and opportunistic cryptococcosis cases, identifying the risk factors and differences in virulence of cryptococcal isolates (Marcia Lazéra, personal communication).

Differences in the global distribution of the genetic diversity suggest that ancient evolutionary events can be detected in the invasion-history of major molecular type VNI. Both the AMOVA and PCA analyses showed that the diversity was statistically significant when the populations were assessed according to continent. In this case, the pairwise *F*_*ST*_ tests showed Africa, North America, and South America had the same groups of STs, while Europe and Asia presented more differentiated subpopulations (see, e.g., map of Figs 3B and 5C). The African-origin hypothesis is supported by different studies [37, 75], in addition to the presence of the highest diversity and the presence of more mating type **a** isolates amongst African isolates. It is very probable that the global VNI population originated in Africa, from where the ancient human migration to Asia [75, 93, 94] and Europe [94] took place as observed in this study. The data presented here and the absence of a statistical difference between the *F*_*ST*_ values from Africa, South America, and North America suggests more recent multiples dispersal events from Africa to the Americas. The clonal characteristics of the VNI population in America in addition to the few evolutionary events observed in the coalescence analysis indicate a more recent evolution.

Analyses using goeBurst and coalescence methods to determine the number of populations in the global dataset, further corroborated the existence of two main clusters. Structure analysis recovered these two clusters, and further divided the major cluster into two subpopulations. Three subpopulations within the *C*. *neoformans* var. *grubii* VNI major molecular type (VNIa, VNIb, and VNIc), including variation in virulence, were recently described using genetic analysis in a South African population [35]. The current study did not find any correlation between the fatal outcome of the patients and the two major clusters analysed (Table 1). This lack of demonstrable variation in virulence is likely explained by the close genetic relationships of the *C*. *neoformans* subpopulations studied.

The coalescence genealogy showed that the two main clusters diverged from the ancestral VNB around 0.58 to 4.0 million years ago. Arising from a subpopulation mainly restricted to Africa, the *C*. *neoformans* var. *grubii* VNI major molecular type likely split into two main groups around 0.29 to 2.8 million years ago. Results of the structure analysis showed that isolates from Africa, South America, and North America were present in both clusters (Figs 4D and 5C), corroborating the lack of difference, which was found by *F*_*ST*_. In contrast, isolates from Europe were comprised of one subpopulation of the major cluster while those from Asia were comprised of a different subpopulation. As evolution is a continuous process, it is possible that these two clusters within the *C*. *neoformans* var. *grubii* VNI would gain specific characteristics as time passed. Thus, further characterization of these subpopulations within the VNI genotype using more discriminatory techniques such as whole genome sequencing will likely add insights into different virulence traits and clinical outcomes, as has already been demonstrated for *C*. *neoformans* var. *grubii* VNB and for the Vancouver Island genotypes of the sibling species *C*. *gattii* VGII [23, 24, 35].

The persistence of widespread clones (e.g. ST93 in Southeast Brazil; ST5 in Asia) that are stable in space and time may follow the features of clonal evolution. Clonal evolution in microbes has been defined as a result of the absence or restriction of genetic recombination due to two main manifestations: (i) strong linkage disequilibrium and (ii) widespread genetic clustering [95]. Our results fit the two manifestations of this concept as follows: First, the majority of populations and the clone corrected dataset showed statistically significant results for tests of non-random association of alleles at different loci (*I*_A_ and rBarD), demonstrating an overwhelmingly clonal population structure (Table 4). Second, a star-like shape distribution or clustering of the isolates was clearly observed using the allelic profile in the goeBurst analysis (Fig 3B), in addition to the convergent results using the phylogenetic, coalescence, PCA, and structure analyses. Furthermore, only a few recombination events were found for *SOD1* in Africa and *GPD1* in Asia, and three of the seven MLST loci in the overall *C*. *neoformans* var. *grubii* VNI population. The few recombination events that we found are likely to be too rare to break the clonal population structure of *C*. *neoformans* var. *grubii* VNI. One main theoretical consequence of our observation of a widespread clonality is the accumulation of deleterious mutations in the genome, known as Muller's ratchet. This phenomenon would be expected in *C*. *neoformans* var. *grubii* VNI as the majority of the population is of one mating type. However, unisexual reproduction can restore mutant strains of *C*. *neoformans* to wild-type genotype and phenotype, including prototrophy and growth rate, thus reverting Muller's ratchet [96].

Overall the herein presented study highlights a clonal population structure of the *C*. *neoformans* var. *grubii* VNI major molecular type in clinical and environmental isolates from southeastern Brazil using the ISHAM consensus MLST scheme. The southeastern Brazilian isolates revealed a highly clonal population structure and were less variable than populations from other continents, despite representing a limited geographic sampling. The finding that ST93 was recovered from the majority of the patients in this study, which was also associated with high mortality in Uganda [77], would suggest that this genotype may be associated with a higher mortality. From a clinical point of view, despite free ART in Brazil, late HIV diagnosis and disseminated cryptococcosis at admission for HIV treatment are still the major problems, which need to be overcome in order to reduce the high mortality of cryptococcosis.

## Supporting Information

S1 Fig Neighbor-joining (NJ) tree of the global *Cryptococcus neoformans* var. *grubii* VNI dataset using the concatenated sequences of the seven MLST loci (*CAP59*, *GPD1*, *LAC1*, *PLB1*, *SOD1*, *URA5*, and the IGS1 region). The optimal tree with the sum of branch length (0.0435) drawn to scale and measuring the number of substitutions per site is shown. Bootstrap values >50% based on 1,000 replicates are presented close to the branches. The analysis involved 92 nucleotide sequences with 3,992 positions revealing the two main clusters (red = major and blue = minor). The isolates are described according to the sequence type number (ST), followed by mating type (**a** or α) and country of isolation, which are abbreviated according to the alfa-2 code of ISO 3166–1. The colours of each country represent the continent of origin as follows: blue: Europe, brown: South America, green: Africa, orange: North America, red: Asia.
(TIF)

S2 Fig Split decomposition analysis of the concatenated global *Cryptococcus neoformans* var. *grubii* VNI MLST dataset applying the Neighbour-net algorithm using the Kimura 2-parameter model and evidencing the diversity and branching ambiguities attributable to recombination events. The recombination events can be evidenced in the picture by the bridges between each ST. The phi test for recombination implemented in the software SplitsTree showed significant evidence (p<0.0001) for recombination. The STs belonging to the two main clusters identified in the previous phylogenetic analysis were also separated using the split decomposition and are highlighted in blue (minor group) and red (major group).
(TIF)

S3 Fig Number of populations (*K*) inferred by the software Structure harvester. The actual number of *K* = 3 was evidenced for all analyses using the pre-defined subpopulations in **A**) the whole *Cryptococcus neoformans* var. *grubii* VNI population, **B**) isolates assessed according to clinical and environmental sources, and **C**) subpopulations assessed according to continent of origin.
(TIF)

S4 Fig Haplotype networks of each MLST marker of the *Cryptococcus neoformans* var. *grubii* VNI isolates. The ancestral genotype is represented by a square, while circles represent descendant’s genotypes. Brown colours surrounding squares and/or circles represent the allele types (AT) found around the world. Blue circles represent the ATs found around the world but not in Africa, while the green circles represent those found only in Africa. Allele type numbers found in Brazil are highlighted in red. The size of both squares and circles is proportional to the number of ATs found in the expanded dataset. The most variable locus was the IGS1 region, followed by *GPD1* and *SOD1* while the least variable was *CAP59*. Dots on the lines connecting the haplotypes represent the most parsimonious number of mutational steps required to generate the allelic polymorphisms.
(TIF)

S1 Table List of the southeastern Brazilian *Cryptococcus neoformans* var. *grubii* VNI isolates and the isolates obtained from the *C*. *neoformans* MLST database for the extended global dataset, containing the ISHAM-MLST allelic profile, GenBank accession numbers, *URA5*-RFLP major molecular type, serotype, mating type, and ploidy. (XLS)

S2 Table Clinical and laboratory characteristics of the 101 southeastern Brazilian patients included in the study. (XLS)

S1 Dataset XML file of the current global population of *Cryptococcus neoformans* var. *grubii* VNI isolates assuming a relaxed log-normal clock and calibrated using a normal distribution of 4.5 million years ago. (XML)
