# Correction: MLST-Based Population Genetic Analysis in a Global Context Reveals Clonality amongst *Cryptococcus neoformans* var. *grubii* VNI Isolates from HIV Patients in Southeastern Brazil

**DOI:** 10.1371/journal.pntd.0005380

**Published:** 2017-02-15

**Authors:** The PLOS Neglected Tropical Diseases Staff

S1–S3 Figs are incorrectly presented. The image that appears as S1 Fig should be S2 Fig, the image that appears as S2 Fig should be S3 Fig and the image that appears as S3 Fig should be S4 Fig. S1 Fig is omitted from the Supporting Information. It can be viewed below, along with the correct order of S1–S3 Figs and their respective captions.

## Supporting Information

S1 Fig Neighbor-joining (NJ) tree of the global *Cryptococcus neoformans* var. *grubii* VNI dataset using the concatenated sequences of the seven MLST loci (*CAP59*, *GPD1*, *LAC1*, *PLB1*, *SOD1*, *URA5*, and the IGS1 region). The optimal tree with the sum of branch length (0.0435) drawn to scale and measuring the number of substitutions per site is shown. Bootstrap values >50% based on 1,000 replicates are presented close to the branches. The analysis involved 92 nucleotide sequences with 3,992 positions revealing the two main clusters (red = major and blue = minor). The isolates are described according to the sequence type number (ST), followed by mating type (**a** or α) and country of isolation, which are abbreviated according to the alfa-2 code of ISO 3166–1. The colours of each country represent the continent of origin as follows: blue: Europe, brown: South America, green: Africa, orange: North America, red: Asia.
(TIF)

S2 Fig Split decomposition analysis of the concatenated global *Cryptococcus neoformans* var. *grubii* VNI MLST dataset applying the Neighbour-net algorithm using the Kimura 2-parameter model and evidencing the diversity and branching ambiguities attributable to recombination events. The recombination events can be evidenced in the picture by the bridges between each ST. The phi test for recombination implemented in the software SplitsTree showed significant evidence (p<0.0001) for recombination. The STs belonging to the two main clusters identified in the previous phylogenetic analysis were also separated using the split decomposition and are highlighted in blue (minor group) and red (major group).
(TIF)

S3 Fig Number of populations (*K*) inferred by the software Structure harvester. The actual number of *K* = 3 was evidenced for all analyses using the pre-defined subpopulations in **A**) the whole *Cryptococcus neoformans* var. *grubii* VNI population, **B**) isolates assessed according to clinical and environmental sources, and **C**) subpopulations assessed according to continent of origin.
(TIF)
