# Supplementary figures and images for: MLST-Based Population Genetic Analysis in a Global Context Reveals Clonality amongst Cryptococcus neoformans var. grubii VNI Isolates from HIV Patients in Southeastern Brazil
Source: PLoS Negl Trop Dis. 2017 Jan 18;11(1):e0005223. doi: 10.1371/journal.pntd.0005223 (PMC5242430; doi:10.1371/journal.pntd.0005223)

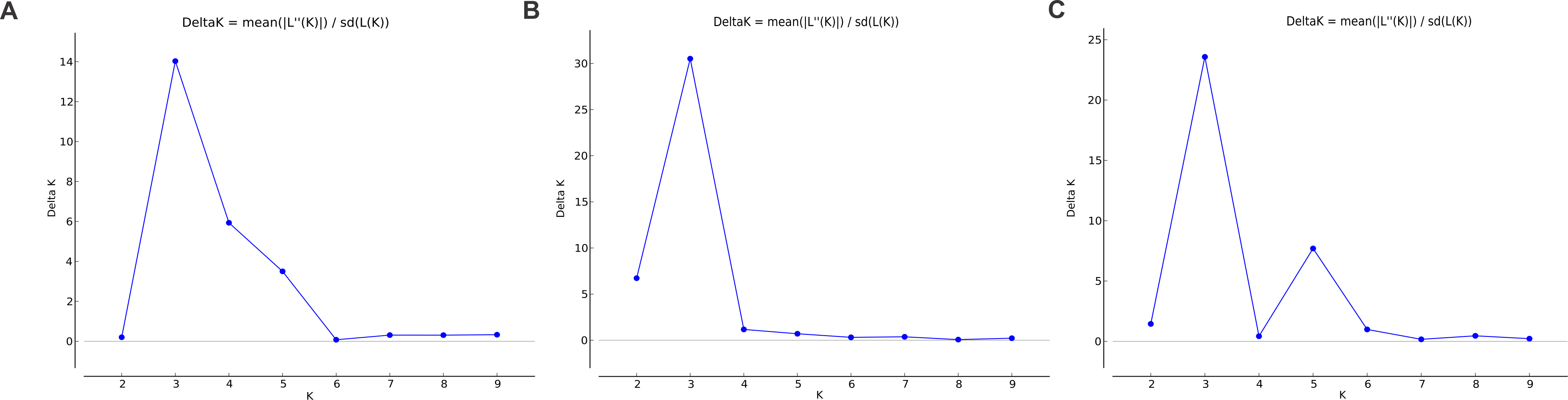

Supplement: S2 Fig — The recombination events can be evidenced in the picture by the bridges between each ST. The phi test for recombination implemented in the software SplitsTree showed significant evidence (p<0.0001) for recombination. The STs belonging to the two main clusters identified in the previous phylogenetic analysis were also separated using the split decomposition and are highlighted in blue (minor group) and red (major group). (TIF) [file pntd.0005223.s002.tif]

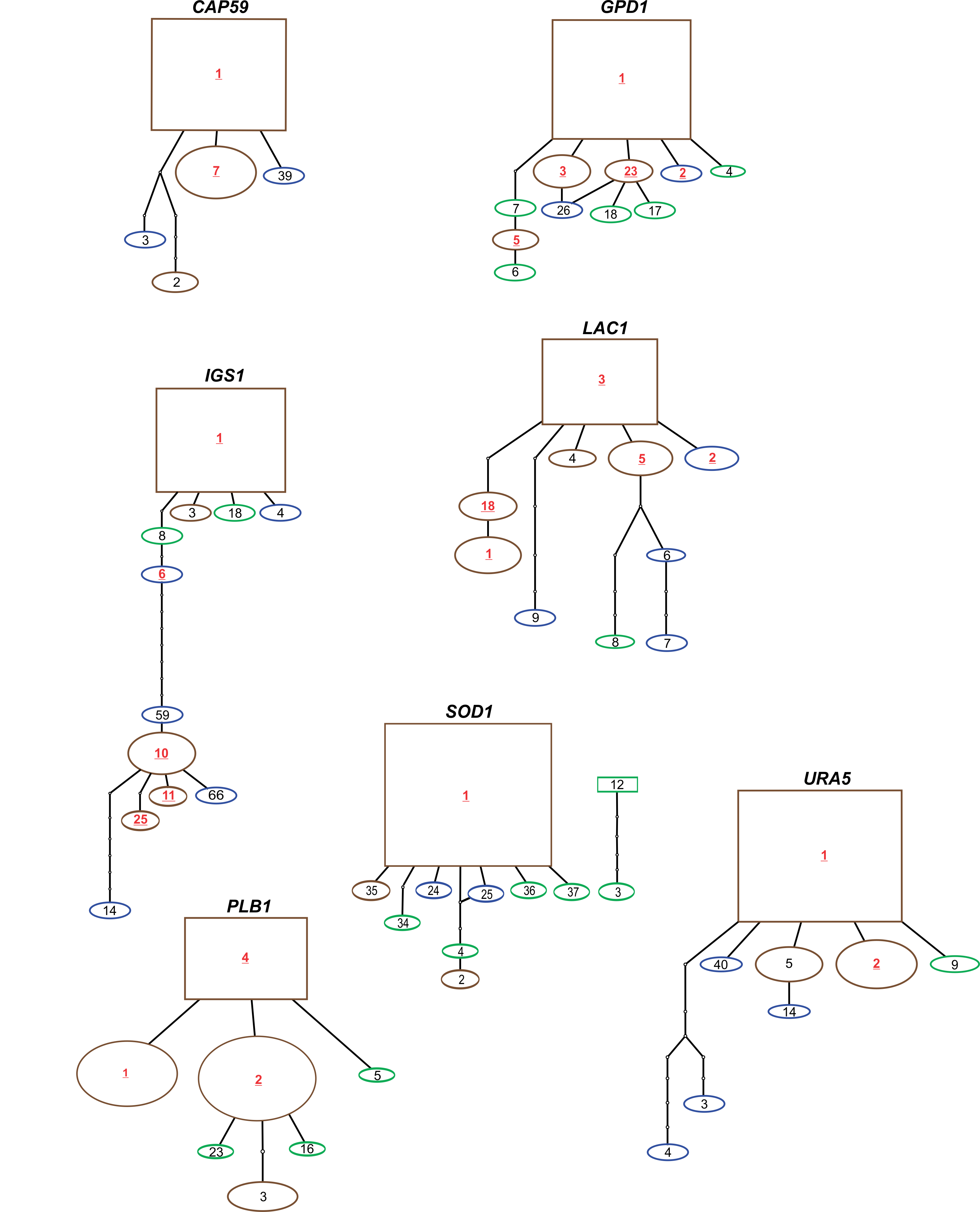

Supplement: S3 Fig — The actual number of K = 3 was evidenced for all analyses using the pre-defined subpopulations in A) the whole Cryptococcus neoformans var. grubii VNI population, B) isolates assessed according to clinical and environmental sources, and C) subpopulations assessed according to continent of origin. (TIF) [file pntd.0005223.s003.tif]

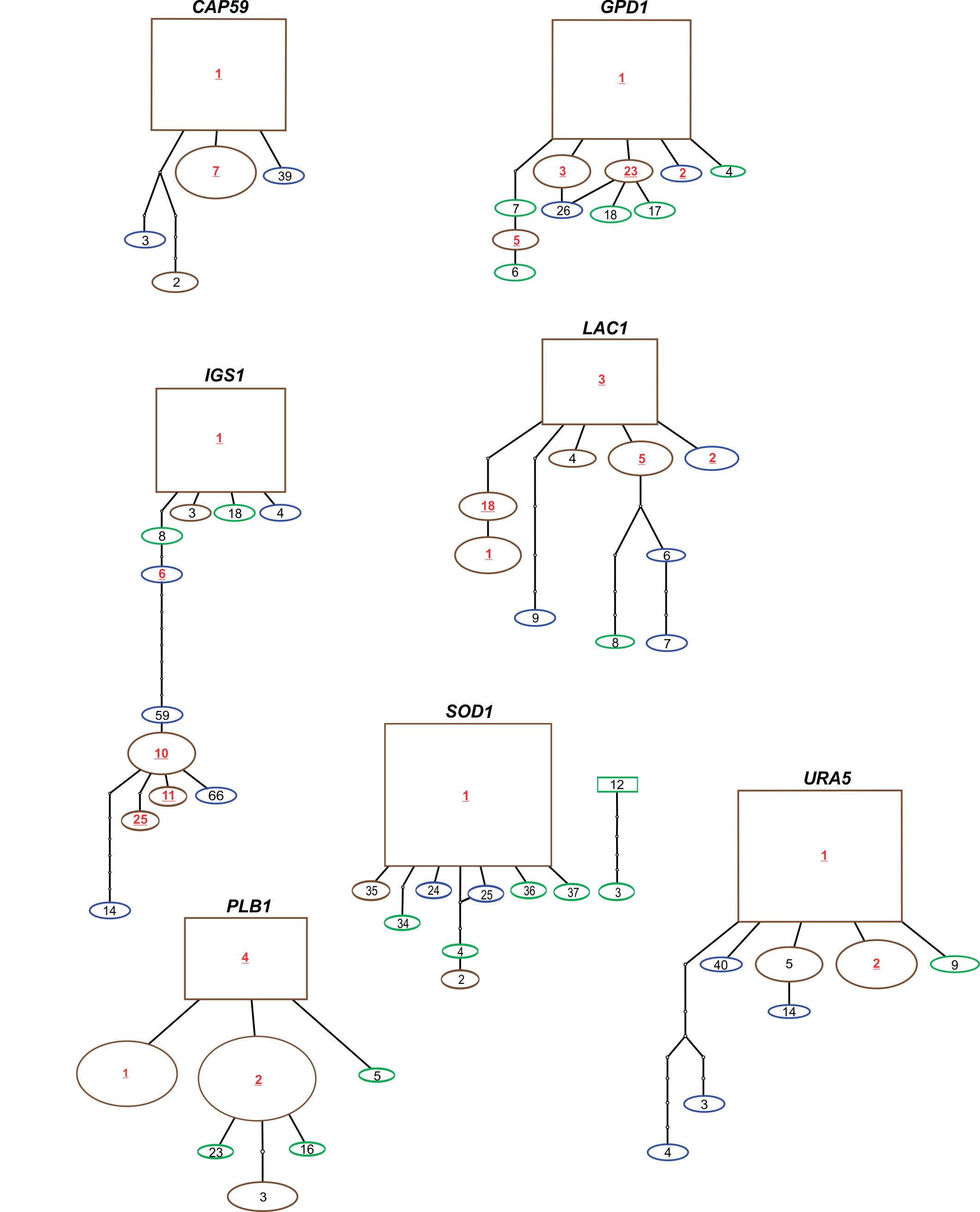

Supplement: S4 Fig — The ancestral genotype is represented by a square, while circles represent descendant’s genotypes. Brown colours surrounding squares and/or circles represent the allele types (AT) found around the world. Blue circles represent the ATs found around the world but not in Africa, while the green circles represent those found only in Africa. Allele type numbers found in Brazil are highlighted in red. The size of both squares and circles is proportional to the number of ATs found in the expanded dataset. The most variable locus was the IGS1 region, followed by GPD1 and SOD1 while the least variable was CAP59. Dots on the lines connecting the haplotypes represent the most parsimonious number of mutational steps required to generate the allelic polymorphisms. (TIF) [file pntd.0005223.s004.tif]
