# Supplementary figures and images for: Correction: MLST-Based Population Genetic Analysis in a Global Context Reveals Clonality amongst Cryptococcus neoformans var. grubii VNI Isolates from HIV Patients in Southeastern Brazil
Source: PLoS Negl Trop Dis. 2017 Feb 15;11(2):e0005380. doi: 10.1371/journal.pntd.0005380 (PMC5310759; doi:10.1371/journal.pntd.0005380)

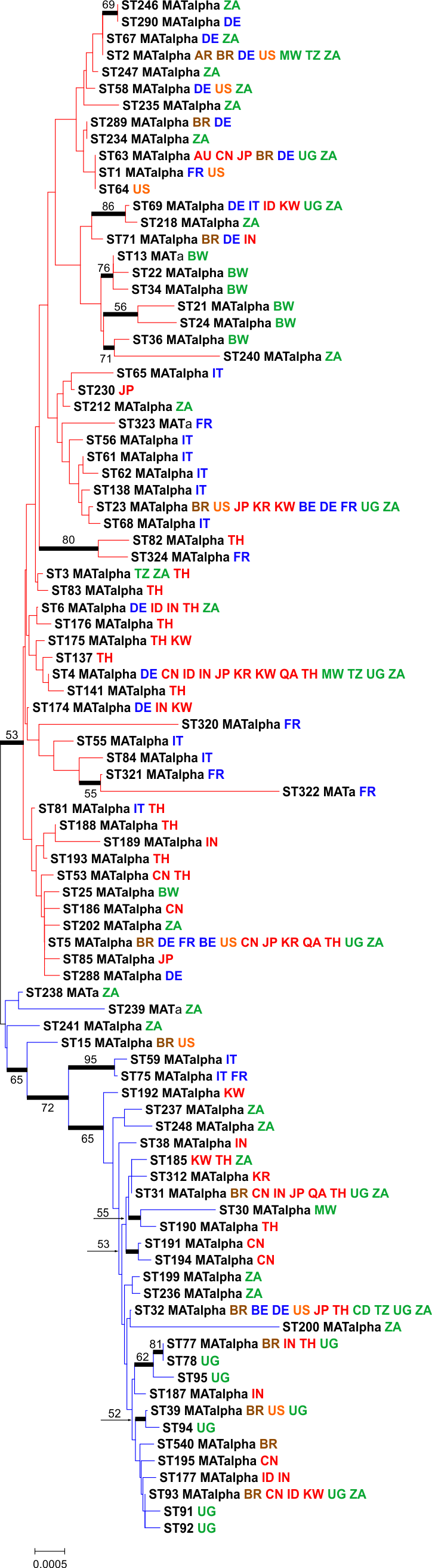

Supplement: S1 Fig — The optimal tree with the sum of branch length (0.0435) drawn to scale and measuring the number of substitutions per site is shown. Bootstrap values >50% based on 1,000 replicates are presented close to the branches. The analysis involved 92 nucleotide sequences with 3,992 positions revealing the two main clusters (red = major and blue = minor). The isolates are described according to the sequence type number (ST), followed by mating type (a or α) and country of isolation, which are abbreviated according to the alfa-2 code of ISO 3166–1. The colours of each country represent the continent of origin as follows: blue: Europe, brown: South America, green: Africa, orange: North America, red: Asia. (TIF) [file pntd.0005380.s001.tif]

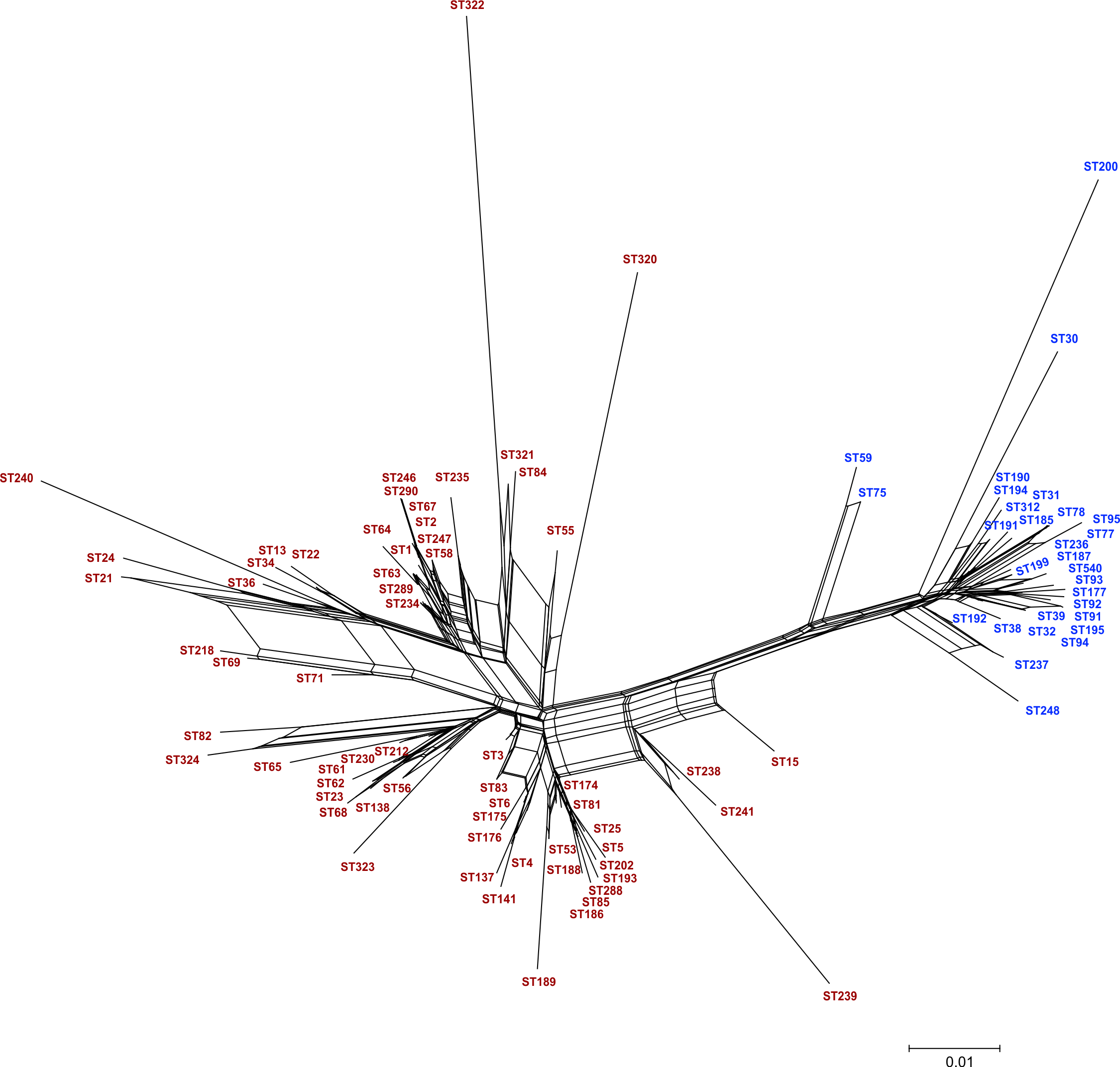

Supplement: S2 Fig — The recombination events can be evidenced in the picture by the bridges between each ST. The phi test for recombination implemented in the software SplitsTree showed significant evidence (p<0.0001) for recombination. The STs belonging to the two main clusters identified in the previous phylogenetic analysis were also separated using the split decomposition and are highlighted in blue (minor group) and red (major group). (TIF) [file pntd.0005380.s002.tif]

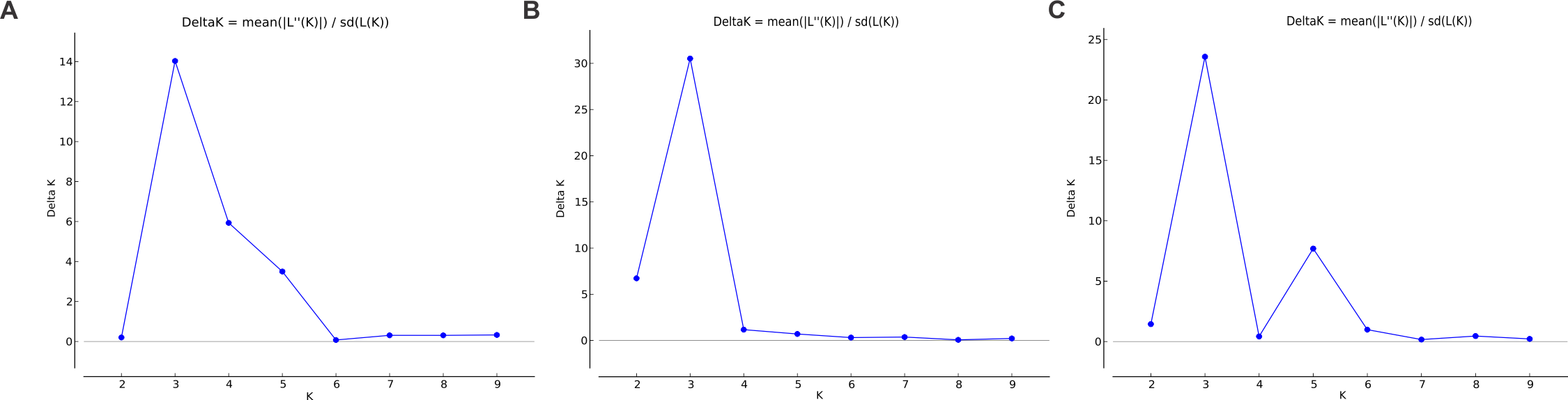

Supplement: S3 Fig — The actual number of K = 3 was evidenced for all analyses using the pre-defined subpopulations in A) the whole Cryptococcus neoformans var. grubii VNI population, B) isolates assessed according to clinical and environmental sources, and C) subpopulations assessed according to continent of origin. (TIF) [file pntd.0005380.s003.tif]
